# Capitalization of Health Promotion Initiatives within French Sports Clubs

**DOI:** 10.3390/ijerph18030888

**Published:** 2021-01-20

**Authors:** Aurélie Van Hoye, Stacey Johnson, Fabienne Lemonnier, Florence Rostan, Laurianne Crochet, Benjamin Tezier, Anne Vuillemin

**Affiliations:** 1 APEMAC, University of Lorraine, 54600 Villers-lès-Nancy, France; benjamin.tezier@univ-lorraine.fr; 2 LAMHESS, Université Côte d’Azur, 06200 Nice, France; stacey.johnson@univ-cotedazur.fr (S.J.); Anne.VUILLEMIN@univ-cotedazur.fr (A.V.); 3 Department of Health Promotion, Santé Publique France, 94410 Saint-Maurice, France; Fabienne.LEMONNIER@santepubliquefrance.fr (F.L.); Florence.ROSTAN@santepubliquefrance.fr (F.R.); Laurianne.CROCHET@santepubliquefrance.fr (L.C.)

**Keywords:** health promotion, settings-based, capitalization, sports clubs, complex interventions

## Abstract

The settings-based approach to health promotion within sports clubs is a growing field of research. Evidence of health promotion intervention effectiveness in scientific literature is scarce, and little is known about their implementation mechanisms. The present study explores how promising health promotion interventions in eight French sports clubs are developed, and how the health promoting sports club’s intervention planning framework is applied. A method to collect Experiential Knowledge in health promotion was used, based on two iterative interviews to analyze intervention mechanisms and completed with document analysis. A deductive analysis using the health promoting sports club intervention planning framework was then undertaken. Among the 14 evidence-driven strategies, 13 were implemented in sports clubs (min = 9; max = 13). Policies were not targeted by any of the interventions. Key competencies of the managers of these health promotion interventions were identified: (1) having a deep understanding of the public and environment, (2) acquiring a high capacity to mobilize internal and external human resources, (3) possessing communication skills and (4) having an ability to write grant applications. By using evidence-driven strategies and intervention components, sports professionals can use this experiential knowledge to create successful and sustainable interventions.

## 1. Introduction

Beyond the benefits of sport practice [1], research has shown that health promotion (HP) activities implemented by sports coaches decreased drop-out intentions, increased enjoyment in sport, as well as self-reported quality of life, among youth and adults [2,3]. According to the World Health Organization (WHO), HP designates “the process of enabling people to increase control over and to improve their health” [4]. To develop this process, the Ottawa Charter [4] pointed out the importance of a settings-based approach [5] in HP, defined as “the place or social context in which people engage in daily activities, in which environmental, organizational and personal factors interact to affect health and well-being” [6]. As researchers have called for multi-level and multi-strategy approaches to health problems [7], the settings-based approach highlights the importance of the whole system in a setting [8], as well as the important potential that sports clubs have to promote health, but it won’t happen in itself [9]. Sports clubs have been defined as “private, non-profit organizations formally independent of the public sector, including volunteer members and a democratic structure, having sport provisions as their main aim” [10], although admittedly, many sports clubs also receive partial financing or support from public sources [11]. Sport clubs are interesting settings from a public health perspective, for several reasons: (1) they welcome a diverse population, across socio-economic status and lifespan [1], (2) the voluntary nature of sport participation provides an opportunity for health education, (3) previous studies have highlighted the benefits of health promotion, such as increased membership or improved sport participants’ health [12]. Nevertheless, recent results have shown that traditional sports clubs are no longer a favorite setting for adults to exercise for health benefits [13]. Thus, working on health promotion could help sports clubs to reinvent themselves in relation to health. In sports clubs, the settings-based approach has been adapted to include determinants (social, organizational, environmental, economic) active at three levels: the macro- (overall policies and orientations of activities of a club), meso- (activities of club management) and the micro level (coaches’ activities in guiding, altering or supporting actions of club members) [14]. In other words, within a single sports club, macro and meso levels set out HP preconditions and aims for the micro level [8]. Cross-sectional studies using self-reported measurements to evaluate sports club’s HP initiatives have underlined the development of environmental and social determinants, but less on organizational and economic determinants [15,16,17]. A recent literature review [18] selected 58 articles encompassing 33 unique interventions of HP within sports clubs. The 33 analyzed interventions mainly focused on a single behavior targeting a single group (male sports participants), limiting the understanding of the application of the settings-based approach (e.g., alcohol policies) [19]. The development of the settings-based approach in sports clubs is relatively recent [20] and its empirical application is still questioned in peer-reviewed articles [18]. Complementarily, previous work has shown that there is limited knowledge on how organizational changes can be implemented to transform sports clubs into health-promoting settings [21,22]. Considering these findings and previous case studies of HP within sports clubs [22], authors have suggested that evidence could potentially be found in grey literature and not only in peer-published articles [23,24]. Nevertheless, few qualitative studies have focused on the mechanisms at play when implementing HP activities. Previous work [16] has shown that (1) sports clubs rarely have written policies, but rather informal oral communication of guidance from management to coaches to sports participants [19], (2) coaches are not aware of the many partnerships managers negotiate to enhance sports clubs’ resources and (3) manager guidance is often oriented towards sport performance rather than health matters [25]. For example, it is more typical for sports clubs to organize sporting events rather than having specific HP interventions. As most of the studies on HP in sports clubs are cross-sectional, previous literature reviews have identified only three effective interventions tested rigorously, worldwide [26], as well as underlined the poor application of settings-based approaches in sports clubs [18], more knowledge is needed specifically on how sports clubs implement HP. In 2020, the health promoting sports club framework was created [27], describing 14 strategies with 55 interventions components, formulated from published evidence-driven guidelines [18]. This framework offers a novel opportunity to understand the health promoting sports club approach with practical implementation guidance. To document organizational changes, the present study analyzes French sports clubs’ HP initiatives to: (1) question how sports clubs develop HP interventions, (2) analyze how the health promoting sports club intervention planning framework, particularly the 14 strategies and 55 interventions components [27], is applied within sports clubs and (3) document elements of transferability from one HP intervention in a sports club into another sports club.

## 2. Materials and Methods 

This is a practice-based research study, on how interventions work in real life [28], in this case grassroots French sports clubs [29,30]. The method has been developed by a national health promotion working group, called Experiential Knowledge in HP (CEKHP) [31], in order to capitalize, collect and circulate knowledge gained from the experience of actors running interventions and to share this within the French health promotion community. CEKHP includes a framework for reporting key mechanisms that influence health promotion intervention outcomes: context, partnership, key steps, barriers and levers, ethics, theoretical foundations and transferability [31]. The present work uses a multiple case study design [32], based on qualitative and iterative methods with four steps: (1) document and website analysis, (2) semi-structured interviews with intervention managers, (3) expert analysis of interventions and (4) validation by intervention managers. Case study research is intended to capture the complexity of the object of study [32], here HP interventions within sports clubs. The multiple data collection is shaped by context and emergent data, with the aim of exploring a real-life, contemporary bounded system over time, through detailed, in-depth data collection involving multiple sources of information [32]. The document and website analysis served as a basis to question the intervention mechanisms and strategies during the interview, helping the interviewer to have a basic understanding of the intervention to support the subsequent in-depth analysis.

### 2.1. Research Settings

Eleven French grassroots sports clubs were identified through researcher networks, multiple foundations (French National Olympic and Sports Committee, Agence pour l’éducation par le sport, Fondaction du football) and sports federations. Sports clubs were selected, because they specifically implemented HP interventions using a setting-based approach, with minimum criteria being to: (1) implicate more than two partners, (2) work on a multi-level strategy (managers, coaches, participants) and target different health determinants (social, organizational, environmental, economic), as well as (3) have at least one year of implementation [27]. Interventions are defined in a wider sense as, “to disturb the ‘natural’ order of things or a foreseeable sequence of events” [33]. In addition to the above criteria, sports clubs had to be affiliated to the French National Olympic and Sports Committee and officiate at grassroots levels. From the 11 originally selected clubs, eight completed the three-step data collection process (Table 1) while the other three only participated in initial interviews. Among these three, one withdrew before the second interview due to lack of time and two were rejected as they did not meet study inclusion criteria (one not yet having a full year of implementation and the other was not affiliated with the French Olympic Committee). 

### 2.2. Procedure

The study was registered with the Commission Nationale de l’Informatique et des Libertés (CNIL). Ethical standards were maintained throughout the study, written consent was secured from sports club directors and key informants. All participants were identified with a code number to protect their identity. No individual data on intervention managers was collected, except their position within the club and employment. Different steps were undertaken: (1) an initial interview to describe the intervention, (2) document analysis, (3) intervention mechanisms and stakes analysis. Step 1: After an initial contact email to the sports club’s intervention manager, a short phone interview (conducted by AVH, FL or FR and LC) helped to (1) document the intervention and ensure fit with inclusion criteria, (2) collect documents and the club’s website address for intervention comprehension and (3) transmit all ethical documents, including informed consent and an intervention information letter. Step 2: Analysis of documents and the intervention was undertaken by the interviewer, to gain knowledge about the intervention and its mechanisms, as well as prepare the second interview. Step 3: a second in-depth interview took place, lasting from 1 to 3 h, to collect more information on the intervention mechanisms and stakes. After the transcription and coding process, the transcribed interview and analyzed data were validated individually by each intervention manager, through email exchanges.

### 2.3. Measurement

Based on the interview guide created by the CEKHP method [31], two semi-structured interview guides were developed by the research team: the club identity card, including the context of the HP action, partnerships and stakeholder implications and evaluation (step 1) and an extended interview guide for an in-person interview, including the in-depth description of the intervention (theory, guidelines, strategies, key moments or experiences, etc.), evaluation, transferability, key mechanisms and required competencies (step 3) (Supplementary File S1). 

### 2.4. Data Analysis

Qualitative data from all interviews were digitally recorded, fully transcribed and analyzed using a paper and pencil approach, to accomplish the organization and coding process. Each sports club’s transcription and collected documents, including information gathered from their website, were read twice to underline key ideas. Then, the Health Promoting Sports Club (HPSC) intervention planning framework [27] was used to analyze interviews and accompanying documents (sports clubs’ regulations, educative project, submitted funding application). The deductive coding approach [34,35] was applied to identify which of the 14 strategies and underlying 55 intervention components from the HPSC intervention planning framework [27] were cited and how they were implemented, by categorizing the quotes into a strategy and linking it with an intervention component. To complement the qualitative analysis, AVH recorded if each intervention component was implemented or not in the sports clubs. This allowed for quantitative data comparison across the eight sports clubs regarding which strategies and intervention components were implemented. Identity cards were individually coded by AVH with a second researcher, SJ, verifying the categorization of each coded intervention component. Then, each full transcription, identity card and intervention component results were sent to the respective intervention manager for verification and validation.

## 3. Results

Among the 14 evidence-driven strategies [27], 13 were implemented in sports clubs, with low variability (minimum of 9; maximum of 13). Policies were not targeted by any of the interventions. Results are presented for each of the 13 evidence-driven strategies. Of the 55 total intervention components (ICs) [27], only eight were not used by the sports clubs. One from motivation, three from participative approach, two from planning and two from policies. Although one of the smaller clubs (324 members) with no paid HP staff, Club 4 implemented 13 of the strategies and used 31 ICs within their project. While Club 1, with the second largest membership and three paid HP staff, implemented 12 strategies but used the highest number of ICs (32). Club 8 had the highest membership base (12,000) and some paid HP staff (1.5), yet implemented the least number of strategies (9) and ICs (14). Both these clubs are multisport. Club 5 & 6 also implemented 12 strategies and used almost half of the total ICs (12 & 24, respectively). Both of these are soccer clubs with less than 1000 members and only Club 5 has a paid HP staff member. Club 7 implemented 11 strategies with 24 ICs, this was the smallest club with only 200 members and no paid staff to implement the health promotion project. Finally, Clubs 2 and 3 both implemented 10 strategies and 18 ICs. While Club 2 has 1000 members and 1 paid HP staff, Club 3 only has 310 with no staff specifically paid for HP. See Table 2 for categorization of intervention components used by each club. See Table 2 for categorization of intervention components used by each club.

### 3.1. Communication (COM)

In the communication strategy, only two sports clubs developed a communication plan (COM1), three created a single message, a slogan (COM2) for the intervention such as “Your safety, our priority” (Club 5), five communicate within the sports club on HP (COM3), seven communicate with the external community on HP (COM4) and three ensured communications with partners (COM5). For example, one intervention manager explained: “But it’s true, we don’t do a 4 by 3 campaign, we communicate through our website, on social networks and through doctor’s offices” (Club 2). Another stated that the first objective was to: “popularize the project within the club in a very, very local way, so well, I’m lucky enough to be able to create video or visual content quite easily, because of my professional activity, so I highlight that a little bit via the club’s Facebook and their website, we made the digital version of our handbook available for free of course, so that allowed us to communicate with the community, parents and other groups, who talked a little bit about all this, it made a little buzz internally” (Club 6). Moreover, communication is the strategy where intervention managers estimated having learned the most, because it is not part of their sport management degree: “The fact of managing the department from A to Z, yes in terms of communication, I discovered a lot of things that we don’t have in our Adapted Physical Activity (APA) training, there is no communication, marketing, partnership, there is some financial management but, I learned about it in more detail although we had concepts” (Club 8). Only Club 1 targeted all five communication intervention components while Club 3 did not target any of these intervention components.

### 3.2. Dynamic (DYN)

In the dynamic strategy, one sports club worked specifically on the feeling of belonging in the club (DYN1), where seven clubs considered how the targeted public related to their social and physical environment and the community (DYN2). For example, an intervention manager explained: “it has to do with the sedentary lifestyle of these isolated or geographically segregated women, so I think that’s the number one issue, the condition, the distance they travel per day in their immediate environment, speaking of health. After that, there are all the axes added to this, the psychological, physical, mental health” (Club 7). A second intervention manager explained: “With this project, we feel useful and we give back a little bit to the community in which we work, it is a way for us to participate in a collective life” (Club 1). While Club 7 targeted both intervention components, Club 8 used none.

### 3.3. Education (EDU)

Among the education strategy, three sports clubs considered support for coaches to actively engage in acquiring HP skills (EDU1). Five sports clubs tailored the support they provide on HP to their coaches (EDU2). Seven sports clubs provided resources for coaches to support each other to promote health (EDU3). One intervention manager explained: “*In this case, in fact, we have done more internal training, whether by sociologists, sports psychologists or sports doctors, to try to understand how a child develops and the impact that diet or lack of sleep can have*” (Club 3). Three sports clubs implemented all three education intervention components.

### 3.4. Experience (EXP)

Six sports clubs based their interventions on the identification of previous experiences in HP (EXP1), six considered organizational readiness (EXP2), three incorporated the reasons for commitment towards HP (EXP3) and one focused on the quality of commitment (EXP4). For example, one sports club started with a health commission before launching the intervention: “*There was already a desire to bring the health side to the club, there was already a sports health commission that was created by the current general secretary and the president who are very conscious of health, because the general secretary is the former director of a rehabilitation center so he has always worked in health and the president was the vice-dean of the faculty of sport; he is now vice-president of the university. So, in fact, they already had a network by word-of-mouth, they created a commission of doctors with the idea of proposing prevention activities for the members*” (Club 8). Another sports club based their actions on observations from the main coach: “*I’ve been taking care of the senior team for seven or eight years. In fact, we noticed that in our daily practice, many, many injuries could have been avoided if, in fact, there had been a message passed on beforehand, hence the idea of carrying out preventive actions to be able to pass on this message and stop having small pathologies of the sportsman on a daily basis which requires sports interruptions and penalize the teams concerned*” (Club 4). No sports club implemented all four intervention components.

### 3.5. Feasibility (FEAS)

Two clubs regularly reviewed financial resources used (FEAS1) and five sports clubs regularly reviewed the human resources being used (FEAS2). Four sports clubs reviewed the sports club’s capacity to undertake the actions required to achieve their goals (FEAS3) and three sports clubs reviewed the time dedicated to achieving the goals (FEAS4). For example, an intervention manager explained: “*We saved time, because we already made a financial commitment for 2020–2021, it was recorded, which was not the case last year, so the actual problem is how long we’ll be able to keep two schools, if it’s done in other schools, other services will be required*” (Club 1). No sports club implemented all four feasibility intervention components.

### 3.6. Goals (GLS)

All sports clubs defined the goals of their HP intervention (GLS1), but only three were formally written (GLS2), one wrote the goals in a positive sporting language based on the sports club’s culture (GLS3) and only four clubs took inclusivity into account while defining the goals (GLS4). For example, one sports club integrated the following statement into their regulations: “*The purpose of the association is to contribute to the physical and cultural development of its members through the practice of football (soccer) and to create bonds of friendship and solidarity among them through the educational and social nature of this sporting activity. To initiate and/or carry out any action in favor of social integration through sport as well as in favor of the development of citizenship, civic morality, the fight against violence, respect for the environment, the fight against discrimination, incivility, health education and solidarity*” (Club 5). No sports club implemented all four intervention components.

### 3.7. Mobilization (MOB)

Two sports clubs mobilized ‘champions’ for their HP interventions (MOB1). One sports club asked a professional player to give her ‘tricks’; the intervention manager explained: “*It’s nice to say what you want, if it’s not legitimized and validated by the testimony of a prominent person, a figure, personality, professional player, it will have less weight than if it’s confirmed by someone who knows what they’re talking about*” (Club 6). Four clubs mobilized experts in HP (MOB2) and seven identified a responsible party within the sports club (MOB3). No sports club implemented all three intervention components.

### 3.8. Monitoring (MON)

Six sports clubs reviewed small improvements achieving HP goals (MON1), four reviewed all HP activities undertaken by the sports club (MON2). Five clubs reviewed the short-term effects (MON3) and three reviewed the long-term effects (MON4) of the HP actions. For example, a intervention manager explained: “*It’s been a year since we’ve designated a player as a health referent, we haven’t done it, and I have players who are today 17 years old and who I had when they were 13 or 14 years old, they’ve kept that reflex of leading the warm-ups, pre-game stretches, post-game stretches and remind the team to hydrate, so that’s a great victory*” (Club 4). One sports club reviewed its HP policies (MON5). One sports club implemented all five monitoring intervention component and one did not implement any. Intervention managers have difficulties finding time and may not have the competency to evaluate their intervention effectiveness, outcomes and mechanisms: “*And that’s something do because it’s true that at the end of the season, it’s always a race and I’ve moved on (from the project) and so we didn’t capitalize too much on that, and again, I didn’t want it to be like an evaluation, like it’s going to be graded*” (Club 6).

### 3.9. Motivation (MOT)

Four sports clubs fostered positive interpersonal stakeholder relationships (MOT1) to improve HP. Four clubs took coaches’ skills to manage situations into account (MOT2). For example, in one sports club, the intervention manager explained that there is a specific profile to coach their players: “*It’s already part of the management skills. It’s not so much a technical skill, since these exercises are the equivalent of what we can do with children that we will transpose to adults, but it’s really the capacity for guidance and observation. To say to oneself: “Okay, I’m going to give a coordination exercise. John Doe does it well. Jane Doe can’t do it, why?” And quickly think, “how do I modify the exercise?*”” (Club 2). Three sports clubs took coaches’ motivation for coaching and future expectations into account (MOT3). Two sports clubs strengthened coaches’ autonomy to promote health (MOT4). For example, an intervention manager explained: “*At the beginning of the season, the first workshop was dedicated to educators, to teach them the right skills (whether on warm-ups, stretching, recovery, nutrition, physical preparation), before the age of 16, so that they, themselves, can be the relay in the field on a daily basis with the children*” (Club 4). No sports club implemented all five motivation intervention components and no sports club worked on strengthening coaches’ sense of ownership of the sports club (MOT5).

### 3.10. Participative Approach (PAP)

Although six sports clubs called attention to all HP actions when interventions were launched (PAP4) none of the clubs recognized specific actions during the intervention (PAP1-3). Three clubs highlighted how HP activities could benefit their sports club (PAP5), such as transmitting information to parents: “*If you send your children to us, they will not only have a technical and tactical approach to soccer, they will also have a civic education in health and sport*” (Club 4). Three clubs included managers, coaches and participants in the decision-making process (PAP6). No sports club implemented all six participative approach intervention components and one sport club did not target any of these intervention components (Club 2).

### 3.11. Partners (PART)

Six sports clubs identified partners for HP (PART1), starting with the question: “We want to do this, how can we get to the end of the project and what are the relative problems we might encounter? Who are the people we could ask to help us?” (Club 1). All of the clubs defined how to collaborate with existing and future partners (PART2) and four sports clubs created a common culture with their partners (PART3). Four sports clubs implemented all three intervention components in this strategy.

### 3.12. Planning (PLAN)

Club 4 included the core goals in its plan (PLAN1), as well as the target population (PLAN2). Two clubs included key steps in implementation plans (PLAN5) and one considered the sustainability of the HP actions (PLAN6). No sports club implemented all six planning intervention components and five did not implemented any of these intervention components. In fact, none of the clubs implemented PLAN3 to include funding sources or PLAN4 to include responsible persons in the implementation process. One intervention manager stated, that two years after the launch: “*We’re in the process of writing something that can be replicated, in any case, we’re working on a project called ‘Fight Anti-discrimination’, in which projects like this will have an emphasis on spreading and multiplying*” (Club 7).

### 3.13. Policies (POL)

None of the sports clubs targeted the policy strategy. In different clubs, despite the implementation of actions, policy writing and planning is still lacking. The intervention manager from Club 8 stated: “*No, the statutes have not been changed, but the club’s development is very health-oriented, it’s very much linked to health and social issues, because the president takes this approach. And the members hear a lot about sport and health*”.

### 3.14. Resources (RES)

Three sports clubs reviewed available financial resources (RES1) and six clubs reviewed their human resources (RES2) to invest in HP. For example, an intervention manager explained: “*We are in the process of developing a financial partnership because until now, we have limited ourselves to private partnerships such as cash donations, but they are not official partners they are simply sponsors who are interested in where they invest money*” (Club 3). Another explained that the life cycle of the intervention is often based on voluntary human resources: “*You can see it in the balance sheets, I think there was six months or a year of hesitation, and the projects are starting up again, it’s also starting up again because it’s up to people, like a lot of projects. We are aware that this also raises questions about the sustainability of the project, if we both leave [the two project managers], it will be very complicated*” (Club 7). Two clubs reviewed current skills and knowledge available to promote health (RES3). Club 1 implemented all three resource intervention components while two clubs did not implement any. Another intervention manager stated: “*No, I don’t see how it can stop, even with little means, we can do it, even if only the manual. At the end of the day, if you don’t have the means, neither human nor financial, you can always involve the educators who say: You continue to develop the Federal Educational Plan”* (Club 5).

## 4. Discussion

The diversity of sports clubs in regard to size, paid staff to undertake HP projects and type of sport (see Table 1 for details) has shown that implementing HP in sports clubs is feasible [18]. The present study questioned eight promising HP interventions of various sports clubs in France to understand how key strategies and intervention components of the HPSC intervention planning framework [27] were implemented. By implementing at least 9 of the 14 evidence-driven strategies from the HPSC intervention framework [18,27], these sports clubs have reinforced the call of many authors highlighting that evidence of settings-based approaches within sports clubs can be found in the grey literature [36]. In other words, the experiential knowledge collected in the present study has filled a gap providing proof for the feasibility of the health promoting sports club approach.

In regard to sports club characteristics, a previous literature review identified limitations regarding the target population, where male team sport participants were the primary target and interventions were only implemented at the sports participant level [18]. The present work has shown a broad diversity of targeted populations (from youth to vulnerable to elderly) and implicated different levels (managers, coaches, participants), as well as including at least two partners in their interventions. These findings reinforce the hypothesis of the applicability of the settings-based approach to sports clubs, as its key characteristics encompass multi-level and multi-strategy interventions in sports clubs [7,18,27]. These findings also raise question about the paucity of published studies on HPSC interventions, which could potentially be explained by the issue of a lack of measurement of health promoting sports clubs, as the impact and implementation of multi-level and multi-strategy interventions is complex to evaluate [14]. The inclusion of 4 soccer clubs among the 8 was explained by the support provided by the soccer federation and directives through the federal education project, providing guidelines for HP, as well as a specific foundation (fondaction du football) to promote sports clubs which develop social responsibility actions. This shows that having guidelines and regulations from sports federations seems to support sports clubs’ engagement in HP [15,37]. 

As interventions have demonstrated, many are effective at targeting a single behavior [26], but the settings-based approach encourages a broader ‘umbrella’ approach. An encouraging result in the present work was that every sports club prioritized a specific thematic (e.g., citizenship, education) or a particular behavior (e.g., healthy eating), yet kept in mind that the HP activity was their first step to a more comprehensive, holistic and participative approach to health [38]. These findings are interesting in regard to the generalization and transferability of the behavioral approach to HP in sports clubs, where focusing on a single behavior could be considered a preliminary step for the settings-based approach [5]. Intervention managers also expressed that their motivation was based on the idea that sports clubs have an important role to play within their communities [39], which aligns with the community health perspective of the settings-based model to create a social context favorable to health.

The CEKPH method was developed to collect and disseminate experiential knowledge about health promotion interventions by creating unique tools such as interview or user guides [31]. The use of the HPSC intervention framework [27] to analyze the collected data, which aligned with the tool created by CEKPH has shown that this framework can serve as a theoretical basis for intervention mapping [40] and settings-based intervention development in sports clubs, with club managers validating the classification after the interview process. 

Interestingly, deepening previous findings [16], many necessary strategies, such as education and goals, implicating written policies, implementation planning (including funding, target population, etc.), as well as evaluation are still lacking in sports clubs, but this does not refrain the actions from taking place. By considering the implemented intervention components, the barriers previously described in the literature, such as lack of policies [16], as well as the classical sequence of project implementation in health promotion [41] does not seem to be necessary in order to implement HPSC interventions in sports clubs. Nevertheless, longitudinal analysis on the project development and long-term effects are needed to solidify these findings.

Two key competences of intervention managers were identified as (1) being able to observe and adapt the interventions to the sports club and its targeted population (by using sports games, specific language, knowing individual life stories, understanding the institutional context of their sports club and surroundings) and (2) being able to find resources (by inviting already known voluntary expertise or by finding specific funding options or partners). Learned competencies during the individual club interventions included: communication (both internal and external), to be able to disseminate the intervention’s specifics, being recognized by other sports clubs and partners and intervention application writing (to increase funding and recognition). Competences that intervention managers wanted to acquire included evaluation, to be able to produce “more objective” evaluations about their interventions, rather than informal discussions. Interestingly, and in opposition to intervention management in HP [42], sports clubs started their interventions in very different ways, some by finding a partner to provide resources, others by identifying a health problem in their sports club, while others used opportunities to offer new services. Depending on the position of intervention manager, some received only passive recognition by the board on the intervention while others were fully supported with the intervention being written into regulations and integrated into the sports club’s activities. This adds to the findings of a previous study regarding manager’s need for support for HP [25]. In regards to the sustainability of interventions, almost half depended on the intervention manager’s involvement and availability, where the other half could be sustained with human resource changes. This is an important point because HP changes are more visible after three sporting seasons [43], and are a step-by-step long lasting effort for sports clubs, with human and financial resources needing to be reviewed regularly [44]. In the present work, when scheduled, each intervention manager only planned their activities for one sporting season and reproduced and/or enhanced from one season to the next [22]. Although the interviews were undertaken with intervention mangers, other sports club actors could give insight into the planning and implementation process. While the present study embraces the diversity of sports clubs, it was not possible to allocate the difference in strategy implementation to sports clubs’ characteristics, such as size, type of sport or other contextual variables; future quantitative research is needed for this aim.

## 5. Conclusions

The present work highlighted the usability of the HPSC intervention framework [27] to document strategies and intervention components of promising HP interventions in French sports clubs. Among the 14 evidence-driven strategies, 13 were implemented in sports clubs (min = 9; max = 12). Policies were not targeted by any of the interventions. An important take-away from this study is that sports clubs varied their use and order of implementing chosen strategies, some intervention managers even reversed the order and finished with writing the project objectives and planning after having implementing it and having found and consulted partners. Some projects did not even include written objectives or prior planning. Key competencies of HP intervention managers have been identified as: (1) a deep understanding of the public and environment, (2) a high capacity to mobilize internal and external human resources, (3) communication skills and (4) grant writing application skills. This research takes a bottom-up approach by learning from HP activities already implemented in sports clubs to expand knowledge on evidence-driven strategies applied within the HPSC intervention planning framework [27]. Practical implications include (1) the applicability of the strategies and intervention components by sports clubs, in the order they find suitable to their needs to develop a HPSC, (2) the support or training sport or health actors can provide on key competencies or the attention given during recruitment of managers in regard to these competencies and (3) the provision of full CEKHP reports [31] from sports clubs on request.

## Figures and Tables

**Table 1:** Main characteristics of the selected sports clubs.

Club	Sport Offered	# of Members	Location	Intervention Manager Position	Health Promotion (HP) Action	HP Paid Staff	Gender & Age (Years)	Funding Source
1	Multisport	7000	Clermont-Ferrand	Responsible for youth education	After school homework	3	All (≥6)	Private partners, city
2	Track & field	1000	Nice	Responsible for the sport and health section	Elderly sports practice	1	All (≥5)	Health Insurance
3	Soccer	310	La Montagne	Responsible for youth education	Youth health behavior and social health	0	All (≥5)	City, private partners
4	Soccer	324	Biot	Main coach	Injury prevention	0	Males (≥5)	None
5	Soccer	500	Epinal	Sport clubs employee	Citizenship	1	All (≥5)	City, department, private partners
6	Soccer	950	Serris	Responsible for female football practice	Youth healthy eating	0	All (≥5)	one
7	Cycling	200	Lille	Director	Integration through sport	0	Females (≥18)	City
8	Multisport	12,000	Marseille	Responsible for the sport and health section	Sport and health services	1.5	All (≥5)	City, Department, Region, national center for sport development, private partners

**Table 2:** Summary of implemented strategies and intervention components among sports clubs.

Club	COM	DYN	EDU	EXP	FEAS	GLS	MOB	MON	MOT	PAP	PART	PLAN	RES	POL
1	COM1 COM2 COM3 COM4 COM5	DYN2	EDU1 EDU2 EDU3	EXP1 EXP2 EXP3	FEAS1 FEAS2 FEAS4	GLS1 GLS2 GLS4	MOB3	MON1 MON2 MON3 MON4 MON5		PAP4	PART1 PART2 PART3	PLAN6	RES1 RES2 RES3	
2	COM1 COM4 COM5	DYN2	EDU3	EXP1 EXP2	FEAS1 FEAS2 FEAS3	GLS1	MOB1 MOB3	MON1 MON3	MOT1 MOT2		PART2			
3		DYN2	EDU1 EDU2 EDU3	EXP4	FEAS3	GLS1 GLS2 GLS4	MOB2 MOB3	MON2 MON3	MOT3 MOT4	PAP4 PAP6	PART2			
4	COM3 COM4	DYN2	EDU2 EDU3	EXP2 EXP3	FEAS2 FEAS3 FEAS4	GLS1 GLS4	MOB2 MOB3	MON1 MON2 MON3 MON4	MOT1 MOT2 MOT3 MOT4	PAP4 PAP5	PART1 PART2	PLAN1 PLAN2 PLAN5	RES1 RES2	
5	COM2 COM4	DYN2	EDU1 EDU2	EXP1		GLS1 GLS2 GLS4	MOB2 MOB3	MON1 MON2	MOT1 MOT2	PAP4 PAP5	PART1 PART2 PART3	PLAN5 PLAN6	RES2	
6	COM3 COM4	DYN2	EDU2 EDU3	EXP1 EXP2	FEAS2	GLS1 GLS3	MOB1 MOB2 MOB3	MON1	MOT1 MOT2 MOT3 MOT4	PAP4 PAP6	PART1 PART2		RES2 RES3	
7	COM2 COM3 COM4 COM5	DYN1 DYN2	EDU3	EXP1 EXP2 EXP3	FEAS3 FEAS4	GLS1	MOB4 MOB6	MON1 MON3 MON4		PAP5 PAP6	PART1 PART2 PART3		RES2	
8	COM3 COM4		EDU3	EXP1 EXP2	FEAS2	GLS1	MOB3			PAP4	PART1 PART2 PART3		RES1 RES2	

COM-Communication; DYN-Dynamics; EDU-Education; EXP-Experience; FEAS-Feasibility; GLS-Goals; MOB-Mobility; MON-Monitoring; MOT-Motivation; PAP-Participative Approach; PART-Partners; PLAN-Planning; RES-Resources; POL-Policies [27].

## Data Availability

The data presented in this study are available on request from the corresponding author. The data are not publicly available due to collection method and privacy for intervention managers.

## Supplementary Materials

The following are available online at https://www.mdpi.com/1660-4601/18/3/888/s1, File S1: Capitalization interview guide.

## References

[1] Eime R.M., Young J.A., Harvey J.T., Charity M.J., Payne W.R. (2013). A systematic review of the psychological and social benefits of participation in sport for children and adolescents: Informing development of a conceptual model of health through sport. Int. J. Behav. Nutr. Phys. Act..

[2] Van Hoye A., Heuzé J.-P., Van den Broucke S., Sarrazin P. (2016). Are coaches’ health promotion activities beneficial for sport participants? A multilevel analysis. J. Sci. Med. Sport.

[3] Van Hoye A., Johnson S., Geidne S., Vuillemin A. (2020). Relationship between coaches’ health promotion activities, sports experience and health among adults. Health Educ. J..

[4] World Health Organization (WHO) The Ottawa Charter for Health Promotion. http://www.who.int/healthpromotion/conferences/previous/ottawa/en/.

[5] Whitelaw S., Baxendale A., Bryce C., MacHardy L., Young I., Witney E. (2001). Settings’ based health promotion: A review. Health Promot. Int..

[6] Nutbeam D. (1998). Health Promotion Glossary. Health Promot. Int..

[7] Jackson S.F., Perkins F., Khandor E., Cordwell L., Hamann S., Buasai S. (2006). Integrated health promotion strategies: A contribution to tackling current and future health challenges. Health Promot. Int..

[8] Kokko S. (2014). Sports clubs as settings for health promotion: Fundamentals and an overview to research. Scand. J. Public Health.

[9] Geidne S., Quennerstedt M., Eriksson C. (2013). The youth sports club as a health-promoting setting: An integrative review of research. Scand. J. Public Health.

[10] Elmose-Østerlund K., Ibsen B., Nagel S., Scheerder J., Slepičková I. (2017). Social integration and volunteering in sports clubs in Europe. Combining knowledge on sports club policies, sports clubs and members in ten European countries. The Values of Sport: Between Tradition and (Post) Modernity, Proceedings of the 14th European Association for Sociology of Sport Conference, Prague, Czech Republic, 14–17 June 2017.

[11] Scheerder J., Zintz T., Delheye P., Sobry C. (2011). The Organization of Sport in Europe: A Patch-Work of Institutions, with Few Shared Points.

[12] Van Hoye A., Heuzé J.-P., Meganck J., Seghers J., Sarrazin P. (2018). Coaches’ and players’ perceptions of health promotion activities in sport clubs. Health Educ. J..

[13] Eime R., Harvey J., Charity M. (2020). Sport participation settings: Where and ‘how’ do Australians play sport?. BMC Public Health.

[14] Johnson S., Vuillemin A., Geidne S., Kokko S., Epstein J., Van Hoye A. (2019). Measuring Health Promotion in Sports Club Settings: A Modified Delphi Study. Health Educ. Behav..

[15] Meganck J., Scheerder J., Thibaut E., Seghers J. (2015). Youth sports clubs’ potential as health-promoting setting: Profiles, motives and barriers. Health Educ. J..

[16] Van Hoye A., Heuzé J.-P., Larsen T., Sarrazin P. (2016). Comparison of coaches’ perceptions and officials guidance towards health promotion in French sport clubs: A mixed method study: Table I. Health Educ. Res..

[17] Kokko S., Kannas L., Villberg J. (2009). Health promotion profile of youth sports clubs in Finland: Club officials’ and coaches’ perceptions. Health Promot. Int..

[18] Geidne S., Kokko S., Seghers J., Koski P., Lane A., Ooms L., Kudlacek M., Vuillemin A., Johnson S., Van Hoye A. (2019). Health promotion interventions in sports clubs: Can we talk about a setting based approach? A systematic mapping review. Health Educ. Behav..

[19] Geidne S., Quennerstedt M., Eriksson C. (2013). The implementation process of alcohol policies in eight Swedish football clubs. Health Educ..

[20] Kokko S., Green L.W., Kannas L. (2014). A review of settings-based health promotion with applications to sports clubs. Health Promot. Int..

[21] Casey M., Harvey J., Eime R., Payne W. (2012). Examining changes in the organisational capacity and sport-related health promotion policies and practices of State Sporting Organizations. Ann. Leis. Res..

[22] Kokko S., Donaldson A., Geidne S., Seghers J., Scheerder J., Meganck J., Lane A., Kelly B., Casey M., Eime R. (2016). Piecing the puzzle together: Case studies of international research in health-promoting sports clubs. Glob. Health Promot..

[23] Priest N., Armstrong R., Doyle J., Waters E. (2008). Policy interventions implemented through sporting organisations for promoting healthy behaviour change. Cochrane Database Syst. Rev..

[24] Priest N., Armstrong R., Doyle J., Waters E. (2008). Interventions implemented through sporting organisations for increasing participation in sport. Cochrane Database Syst. Rev..

[25] Kokko S., Kannas L., Villberg J., Ormshaw M. (2011). Health promotion guidance activity of youth sports clubs. Health Educ..

[26] McFadyen T., Chai L.K., Wyse R., Kingsland M., Yoong S.L., Clinton-McHarg T., Bauman A., Wiggers J., Rissel C., Williams C.M. (2018). Strategies to improve the implementation of policies, practices or programmes in sporting organisations targeting poor diet, physical inactivity, obesity, risky alcohol use or tobacco use: A systematic review. BMJ Open.

[27] Van Hoye A., Johnson S., Geidne S., Donaldson A., Rostan F., Lemonnier F., Vuillemin A. (2020). The health promoting sports club model: An intervention planning framework. Health Promot. Int..

[28] Hohmann A.A., Shear M.K. (2002). Community-Based Intervention Research: Coping With the “Noise” of Real Life in Study Design. AJP.

[29] Green L.W. (2008). Making research relevant: If it is an evidence-based practice, where’s the practice-based evidence?. Fam. Pract..

[30] Green L.W. (2006). Public Health Asks of Systems Science: To Advance Our Evidence-Based Practice, Can You Help Us Get More Practice-Based Evidence?. Am. J. Public Health.

[31] Laurent A., Ferron C., Berry P., Soudier B., Georgelin B., Gaspard S., Berdougo F., Rush E., Lombrail P., National Committee to Promote EKHP (2020). Valuing experiential knowledge in health promotion: A new method to build up knowledge in France. Eur. J. Public Health.

[32] Stake R.E. (2006). Multiple Case Study Analysis.

[33] Hawe P., Potvin L. (2009). What Is Population Health Intervention Research?. Can. J. Public Health.

[34] Raskind I.G., Shelton R.C., Comeau D.L., Cooper H.L.F., Griffith D.M., Kegler M.C. (2019). A Review of Qualitative Data Analysis Practices in Health Education and Health Behavior Research. Health Educ. Behav..

[35] Stuckey H. (2015). The second step in data analysis: Coding qualitative research data. J. Soc. Health Diabetes.

[36] Kokko S., Selänne H., Alanko L., Heinonen O.J., Korpelainen R., Savonen K., Vasankari T., Kannas L., Kujala U.M., Aira T. (2015). Health promotion activities of sports clubs and coaches, and health and health behaviours in youth participating in sports clubs: The Health Promoting Sports Club study. BMJ Open Sport Exerc. Med..

[37] Meganck J., Seghers J., Scheerder J. (2016). Exploring strategies to improve the health promotion orientation of Flemish sports clubs. Health Promot. Int..

[38] Casey M.M., Payne W.R., Brown S.J., Eime R.M. (2009). Engaging community sport and recreation organisations in population health interventions: Factors affecting the formation, implementation, and institutionalisation of partnerships efforts. Ann. Leis. Res..

[39] Misener L., Misener K.E. (2016). Examining the integration of sport and health promotion: Partnership or paradox?. Int. J. Sport Policy Politics.

[40] Bartholomew L.K., Parcel G.S., Kok G. (1998). Intervention mapping: A process for developing theory- and evidence-based health education programs. Health Educ. Behav..

[41] Fertman C.I., Allensworth D.D. (2010). Health Promotion Programs: From Theory to Practice.

[42] Kokko S. (2014). Guidelines for Youth Sports Clubs to Develop, Implement, and Assess Health Promotion Within Its Activities. Health Promot. Pract..

[43] Lane A., Murphy N., Donohoe A., Regan C. (2020). A healthy sports club initiative in action in Ireland. Health Educ. J..

[44] Casey M.M., Payne W.R., Eime R.M. (2012). Organisational readiness and capacity building strategies of sporting organisations to promote health. Sport Manag. Rev..

