# Supplementary material for: Capitalization of Health Promotion Initiatives within French Sports Clubs
Source: Int J Environ Res Public Health. 2021 Jan 20;18(3):888. doi: 10.3390/ijerph18030888 (PMC7908457; doi:10.3390/ijerph18030888)
Supplement: Supplementary file 1 [file ijerph-18-00888-s001.pdf]

Capitalization Interview Guide

document de travail

Identity card headings

- Themes
- Objectives
- Targeted population
- Strategies used
- Implemented activities
- Funding
- Territory/location of action implementation (rural, urban, suburban)
- Involved partners
- Evaluation indicators

Explored elements

Elements of the context of action (*There is no consensus on the notion of 'context') – there is no single model to describe the context which allows all dimensions to be described.*

- **Nature of the environment: rural/urban/ suburban (collect more detail compared to the ID card)**
- **Starting points/emergence of the action: why choose this action at this point in time?**
- **Description of the general issue of the project in context**
- **Context-related barriers and levers:**
    - At the national level (text- call for project proposals- new legislation- programs- plans)
    - At the regional level (PRS priority- call for project proposals, etc.)
    - At the local level

- At the organizational level
  - Inclusion of the action/project in the organization: stakeholder involvement- inclusion in a global project- existence of related actions, etc.

Partners

- **Nature of formed partnerships**
- **Mobilized skills/skills to be strengthened/missing skills**
- **Distribution and, when needed, development of the roles of the partners involved**
- **Barriers and levers to partnerships and counter strategies**

The action

- **Emergence of the action: actions developed elsewhere, reference readings, specific demands, etc.**
- **Action references: underlying theories, guides to good practice and methods, etc.**
- **Stakeholder involvement procedures**
  - mobilization and level of partner involvement
  - mobilization and level of implicated users/citizens
- **Choice of strategies**
- **Analysis of key moments of action**
  - identification of key moments
  - analysis of 3 key moments
- **Success factors**
  - perception of what was successful in the action
  - elements/factors which fostered these successes: identify 3 elements
- **Barriers**
  - perception of failed elements in the action
  - elements/factors which caused these failures: identify 3 elements

Evaluation of the action

document de travail

- **Method and evaluation tools**
- **Principal results of the evaluation (in terms of results and processes)**

Reproducibility/transferability

- **Perception of the reproducibility in the same or different context**
- **Perception of the conditions of reproducibility/transferability (place, public, moments)**

Project / introduction to the interview:

Within the framework of a research and development project regarding health promotion in sports clubs (PROSCeSS), we would like to identify encouraging experiences of health promotion interventions (actions, initiatives) developed within sports clubs. This project, which we are working on throughout France, is the subject of a partnership between the University of Lorraine, Université Côte d'Azur and Santé publique France.
We have adapted a method and a guide for capitalizing on the data from the experience, built collectively by a national group led by the National Federation for Education and Health Promotion – FNES and the French Public Health Society – SFSP[1]. The aim is to collect as much detailed information as possible on the actions carried out in the field, in order to analyze, edit and share the information in order to enrich practices with the lessons learned from other's experiences, and to strengthen the skills of actors involved.

This information primarily concerns the strategies used in the actions and their key moments. We have already established, with your club, the identity card of the actions taken. The interview will be both voice recorded and notes will be taken. It will take about 2 hours to fully understand and document the actions taken within your club.

The text of this interview will be transcribed and analyzed to sort and organize all the information.

The product of this analysis will be sent to you for validation.

The section on data ownership will be integrated here (charter of the SFSP-FNES practical guide)

document de travail

[1] Fédération nationale d'éducation et de promotion de la santé et Société française de santé publique

Objective: respond to the question: How does the club integrate health into its daily dynamic?

Variables	Question(s)	Follow-up Questions
**CONTEXT OF THE ACTION**		
**Issue of the action**	Which health problem(s) does your action/project address?<br><br>Within your environment how does your project address the problem(s)?	Explore the elements for clarification:<br>- diagnostic or existing data<br>- perception through experience/knowledge of the field (provide examples)
**Starting point/Emergence of the action**	Can you tell me how this project was conceived?<br>(*this question is not to explore content, but rather context, initiators, opportunities*)	Why have you engaged in this action at this time?<br><br>What events/opportunities triggered considering this action?<br>- Opportunities at the national level<br>- Opportunities at the regional level<br>- Opportunities at the local level or within your organization?<br>- An observation / needs / problems within your organization?<br>In what way did you feel the context was favorable when you initiated this action?<br>Were there any obstacles linked to the context, any resistance in the action's environment that made its implementation difficult?
**Position of the action within the organization**	How is this action integrated within the daily life of your organization?	Is it part of the club's community project? With which goals?<br>Is it an extension of another project, and if so, which one?<br>Does your club dedicate human and / or financial resources to this type of project?
**Funding the action**	How is this specific action funded?	Did your club allocate a budget? Human resources? Have your partners contributed to the funding? Have you received a grant?
**THE ACTION**		
**Partners**	What partners were involved in your project/action?	What were the specific skills or competencies of each of them?<br>What was the role of each partner?

document de travail

	Have the roles of the partners changed over the course of the project?	Why, in what manner?
	According to you, which partners/types of partners were missing during the course of this action/project?	Today, for example, if you had to replicate this action, what new skills would you mobilize?
	How would you like the partnership to work?	Possible obstacles to the successful functioning of the partnership?<br>How did you manage the obstacles?<br>Levers for successful operation
**Elaboration of the action**	On which theoretical and practical elements did you base your action?<br>*(This question is to explore the theoretical and practical frame of reference not the context*)	What examples of similar actions inspired you? What practical guides or references have helped you?<br>Could you recommend one or two relevant documents?
**Developed strategies**	How have you defined your target population? What was done to mobilize them?	Detailed description of the relays and supports<br>Why this choice?
	How did you define public participation in your action?	Describe the modalities to associate the different phases of the action
	What action strategies have you used?	Why have you chosen these? What hypothesis have you made?<br>Describe the strategy, the modalities to implement these tools
**Communication around the action / development**	Was a communication/value added strategy carried out?	Describe the strategy, implementation procedures and tools (with whom, how and when).
**Perception of the action**	Please identify 3 key moments of the action. These are the moments when you felt that something happened either to promote the dynamic or to slow it down. These can be moments that you identify as positive or difficult.	Why or what do you think made this moment crucial?<br>How did you overcome the more difficult moments?<br>Explore several dimensions related to the environment, skills, public, choice of strategies, partnerships, etc.
	Please tell me why you consider this action a success?<br>Have you seen any unanticipated positive effects?	Identify three success factors<br><br>Did this action produce benefits that were not included in your original objectives, for example increased member loyalty?
	What have you learned from this action in your professional practice?	If you reproduce this action what would you keep, what would you change?

document de travail

	What competencies have you personally developed?	
**EVALUATION**		
**Evaluation method**	How have you evaluated the action?	Which method and which evaluation indicators did you choose?<br>Who is in charge of evaluation?<br>Was an evaluation document produced? How was it shared and in what format?
**Results**	What do you think are the principle results of the action?	What changes did this action produce?<br>Explore changes in the public as well as in practices and dynamics around the club
	According to you, how has this action contributed to integrate health into the daily dynamic of the club?	Did a manager or beneficiaries question the club's commitment to the project?<br>Does this type of project meet an objective of a community project?<br>If no, is it foreseen to introduce an objective relating to health in this project?
**Reproducibility/transferability**	What conditions are necessary to reproduce this action elsewhere?<br><br>What type of support would you have liked to have when you began this project?	If you had to advise an intervention manager working in a similar context, what advice would you give them to carry out the same action?<br>What are the essential 'ingredients' for good results?<br>What may have made it easier for you when you started the project, what might be helpful to another club interested in carrying out the same type of project?
**Records/documents**	Do you have any documents or writings produced or used in connection with this action that you could provide?	Does an educational project exist? A charter? regulations? press articles? educational support materials?

document de travail
